# A High-Voltage Energy-Harvesting Interface for Irregular Kinetic Energy Harvesting in IoT Systems with 1365% Improvement Using All-NMOS Power Switches and Ultra-low Quiescent Current Controller

**DOI:** 10.3390/s19173685

**Published:** 2019-08-24

**Authors:** Hassan Saif, Muhammad Bilawal Khan, Jongmin Lee, Kyoungho Lee, Yoonmyung Lee

**Affiliations:** 1 College of Information and Communication Engineering, Sungkyunkwan University, Suwon 16419, Korea; 2 Korea Electrotechnology Research Institute, Changwon 51543, Korea

**Keywords:** high voltage harvester, pulse harvester, piezoelectric energy harvesting, triboelectric energy harvesting, low quiescent current, wakeup controller, voltage peak detector, power electronics circuits, step-down converter, wide voltage range

## Abstract

An energy-harvesting interface for kinetic energy harvesting from high-voltage piezoelectric and triboelectric generators is proposed in this paper. Unlike the conventional kinetic energy-harvesting interfaces optimized for continuous sinusoidal input, the proposed harvesting interface can efficiently handle irregular and random high voltage energy inputs. An N-type mosfet (NMOS)-only power stage design is introduced to simplify power switch drivers and minimize conduction loss. Controller active mode power is also reduced by introducing a new voltage peak detector. For efficient operation with potentially long intervals between random kinetic energy inputs, standby power consumption is minimized by monitoring the input with a 43 pW wake-up controller and power-gating all other circuits during the standby intervals. The proposed harvesting interface can harvest energy from a wide range of energy inputs, 10 s of nJ to 10 s of µJ energy/pulse, with an input voltage range of 5–200 V and an output range of 2.4–4 V under discontinuous as well as continuous excitation. The proposed interface is examined in two scenarios, with integrated power stage devices (maximum input 45 V) and with discrete power stage devices (maximum input 200 V), and the harvesting efficiency is improved by up to 600% and 1350%, respectively, compared to the case when harvesting is performed with a full bridge rectifier.

## 1. Introduction

Kinetic energy harvesting has been drawing significant attention in recent years since it can potentially extend battery lifetime or even facilitate energy autonomy for battery-operated IoT systems or portable/wearable electronics [1,2,3]. For kinetic energy harvesting, energy harvesters with periodic excitations, such as cantilever-based piezoelectric energy harvesters, are widely studied since they generate regular periodic energy input, which allows the optimization of the harvesting interface circuit at the resonant frequency. Many state-of-the-art kinetic energy harvesting interfaces [4,5,6] have been proposed for energy harvesters with such periodic inputs at relatively low (<5 V) harvester output voltages (*V_HRV_*).

Recently, high voltage kinetic energy harvesters, such as triboelectric generators (TEG) [7,8] and high-voltage piezoelectric generators (HV-PEG) [9,10], have been actively investigated by the material science research community. These energy harvesters offer high energy density but at a relatively high *V_HRV_* (>5 V). High energy density can be helpful in reducing the system form factor if the high *V_HRV_* can be handled efficiently. In addition, if energy harvesting is realized for energy harvesters that generate irregular output, unlike the cantilever-based energy generators, kinetic energy can be harvested from a wider range of motions, e.g., computer key strokes and knee joint motion, and from new types of generators. For example, a TEG generates asymmetric pulsed output with different positive and negative peak voltages and delay between successive pulses even under periodic excitation [7,8]. Therefore, to achieve efficient energy harvesting from various types of motions and harvesting sources, a harvesting interface that can handle energy inputs with a wide range of voltages and various types of excitation modes is desired.

In recent years, a few harvesting interface designs [11,12,13,14] have been published that can deal with discontinuous energy input with high voltages. However, these designs suffer from a number of limitations:

(1) In high-voltage harvesting interfaces [11,12,13], the power stage consists of a combination of HV PMOS and NMOS. HV NMOS can be driven by output/battery voltage (*V_BAT_*) referenced drivers. Because *V_BAT_* is stable, NMOS inherits a simple driving scheme. On the other hand, HV PMOS requires a high-voltage (*V_HRV_*) referenced driving scheme. Under random input harvesting, stable *V_HRV_* cannot be maintained without additional architecture and power overhead. Thus, unstable high-voltage *V_HRV_* complicates the driving requirements for HV PMOS, which results in significant driving power overhead and also increases the system cost as HV PMOS driving interface consists of HV devices.
(2) Moreover, the standby powers of the prior harvesting interfaces [12,13,14] are 10 s to 100 s of nW, limiting their application for harvesting under excitations with long idle periods.
(3) Furthermore, for the harvesting interfaces implementing voltage peaking event detection, sample and hold comparators are employed [14]. Periodic charging/discharging of a sampling capacitor incurs power overhead, which may lead to inefficient harvesting at low-energy input.

These factors suggest the potential for improvement in discontinuous harvesting interface efficiency by adopting an HV NMOS power stage, lowering standby power by an ultra-low quiescent current wake-up controller (WUC) and reducing harvesting interface active mode controller power overhead by the use of an efficient voltage peak detection scheme.

**Figure 1:**
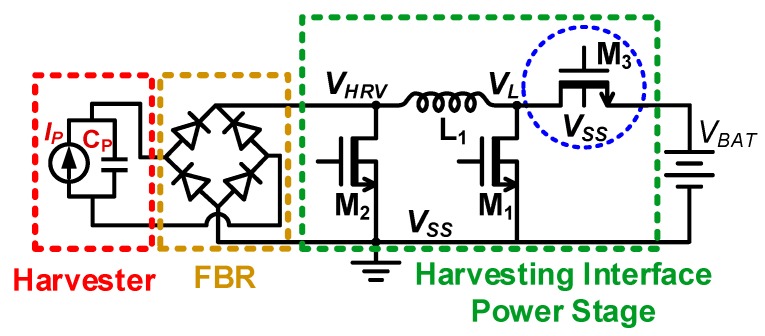
Proposed harvesting interface power stage design.

In this work, a new harvesting interface is proposed to efficiently charge low-voltage batteries (2.4–4 V) with high voltage (5–200 V) PEG/TEG output in continuous as well as discontinuous form. The proposed harvesting interface architecture primarily consists of two blocks: the power stage and the controller. Unlike prior architectures that use high voltage NMOS and PMOS in the power stage, the proposed harvesting interface power stage uses only high voltage NMOS as shown in Figure 1. NMOS with the source terminal at ground (*V_SS_*) potential (M_1_, M_2_) can be directly driven by the *V_BAT_* swing driving signal, but *V_BAT_* source-connected NMOS (M_3_) cannot be driven by the *V_BAT_* swing signal. To drive M_3_, an output (*V_BAT_*) referenced, low-voltage device driving scheme is introduced in this work. NMOS-only power stage implementation eliminates high-voltage (*V_HRV_*) referenced gate drivers. Thus, all drivers can be designed with low voltage devices; which simplifies the driving scheme and reduces the switching loss. Moreover, the high mobility of NMOS charge carriers also improves efficiency by offering a better tradeoff between switching and conduction loss.

To minimize controller power overhead, an event-based controller is implemented. The event-based controller limits controller power overhead by activating most sub-circuits only when energy input is detected; during standby mode, most of the sub-circuits are disabled/power gated.

During standby mode, the event-based controller activates only the wake-up controller (WUC); thus, for efficient harvesting under random excitation, an ultra-low quiescent current WUC is a key building block. Discontinuous excitation may result in long idle periods between successive *V_HRV_* pulses; thus, for efficient harvesting under discontinuous input, the WUC must consume low quiescent current. The standby power consumption (WUC quiescent power) of prior harvesting interfaces [12,13,14] is in the nano-Watts range. To improve harvesting interface standby performance, a new WUC is introduced that consumes only a few pico-Watts quiescent power. Thus, the proposed topology results in a significant reduction in standby power as compared to the prior arts. Moreover, a low voltage rejection filter is also introduced in the proposed WUC to inhibit harvesting from low voltage/energy input, which may result in negative efficiency.

Furthermore, to reduce controller active mode current consumption, a new inverter-based voltage peak detector (VPD) is introduced. The proposed VPD determines the voltage peak by evaluating the slope of *V_HRV_*. The proposed harvesting interface consumes only a few nJ per harvesting cycle, and standby power consumption is in the pW range. Thus, the proposed circuit can efficiently harvest form excitation that results in as low as 10 s of nJ energy per pulse even under discontinuous excitation. To limit the power overhead, all active devices in the controller and drivers are standard low-voltage devices, whereas high-voltage (HV) devices are used only for the power stage and for coupling HV at the low voltage controller.

The remainder of this paper is organized as follows: Section 2 briefly describes the harvester’s maximum energy extraction condition and illustrates the top level architecture of the proposed harvesting interface and its operation cycle. Then implementation details of the proposed interface controller sub-circuits and drivers are presented in Section 3. In Section 4, the results are shown to verify the effectiveness of the proposed approach, and Section 5 concludes the paper.

## 2. Harvester Maximum Energy Extraction Condition and Top Level Harvesting Interface Architecture

### 2.1. Output Capacitive Load Isolation Harvesting

For harvesting energy under periodic input excitation, a maximum power point tracking technique [15,16,17,18] is employed, which tracks the maximum power point at the excitation frequency. However, under random excitation conditions, maximum power point tracking cannot be employed since there is no fixed operating frequency/open circuit voltage for which the harvesting operation can be optimized [13,14]. Therefore, for effective energy harvesting under random excitation, a new scheme for maximizing energy extraction is proposed in [19]. In this scheme, the extracted energy is maximized by isolating the output capacitive load of the harvester during energy/charge extraction. The amount of charge generated by the harvester (*Q*), the capacitance of the load seen by the harvester (*C*), the final output voltage of the harvester at the end of energy extraction (*V*) and the amount of energy generated by the harvester (*E_HRV_*) can be related as:

(1) $$V=\frac{Q}{C}$$

(2) $$E_{HRV}=\frac{1}{2}CV^{2}=\frac{1}{2}\frac{Q^{2}}{C}$$

In the load isolation scheme in [19], a load-isolating transistor is placed at the input of energy harvesting interface, which minimizes the effective capacitance seen by the harvester source during internal capacitor (C_P_) charging. Therefore, assuming *Q* is fixed for a given deformation on the harvester [7,8,9,10], as the load capacitance *C* is minimized, the final accumulated voltage (*V*) is maximized according to Equation (1). This also maximizes *E_HRV_* with maximized *V* or minimized *C* in Equation (2).

The proposed harvesting interface also adopts this load isolation approach; the capacitive load seen by the harvester is minimized as long as the harvester output voltage increases. Only the voltage peak detector (VPD) is enabled during this period, which activates harvesting operation upon voltage peak detection. As the harvesting operation is activated, the harvesting interface transfers energy from the harvester to the inductor and then from the inductor to the battery.

Harvesting interfaces with similar voltage peak detection schemes have been implemented in a few prior works [11,12,13,14] but with a CMOS power stage, i.e., both PMOS and NMOS are required for the power stage. Under random high-voltage input (*V_HRV_*), PMOS driving requires a complex high-voltage driving circuit as described previously, which degrades the harvesting efficiency. To address this challenge, a harvesting interface with an all-NMOS power stage is proposed in this work as shown in Figure 1. The proposed harvesting interface improves efficiency not only by reducing the driving overhead but also by the lowering the conduction loss due to the higher mobility of NMOS compared with that of PMOS.

### 2.2. Proposed Harvesting Interface Top Level Architecture

**Figure 2:**
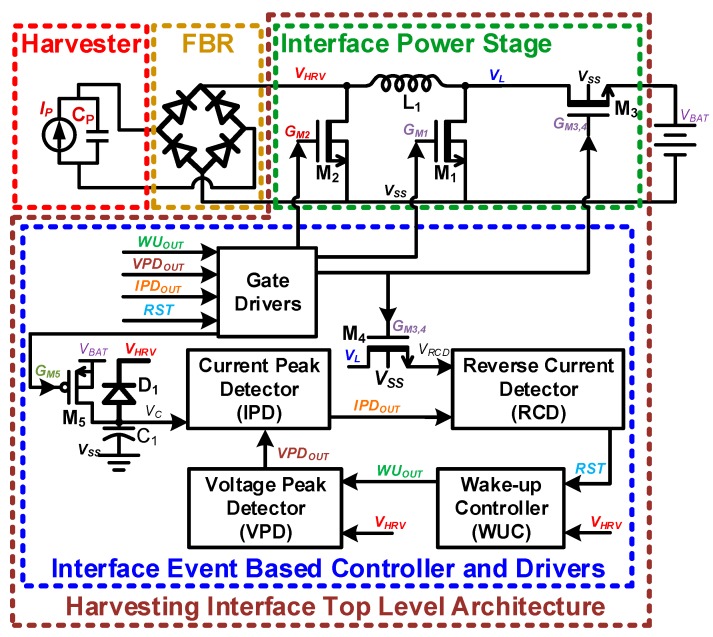
Top level block diagram of the proposed harvesting interface.

A top level block diagram of the proposed harvesting interface is shown in Figure 2. The proposed harvesting interface consists of a power stage and event-based controller/drivers. The controller is further divided into the WUC, which detects harvestable input; the VPD, which detects voltage peaking event; the inductor current peak detector (IPD), which indicates energy transfer completion from harvester source to inductor, by detecting inductor peak current; the reverse current detector (RCD), which indicates energy transfer completion from inductor to the battery, by detecting the reverse current from the battery; and high-voltage interface circuits (gate drivers in Figure 2). An event-based controller is implemented, which means that most of the controller is only activated when energy input is detected. The WUC is the only sub-block that is activated when the harvester is idle; the rest of the sub-blocks are deactivated/power-gated to minimize standby power consumption. The implementation details of the event-based controller are provided in the latter part of this section. A novel WUC and VPD have been proposed in this work, which are described in detail in Section 3. For the IPD and RCD, continuous comparator schemes presented in [20] have been adopted, and the details are provided in supplementary Figure S1.

### 2.3. Proposed Harvesting Interface Operation

**Figure 3:**
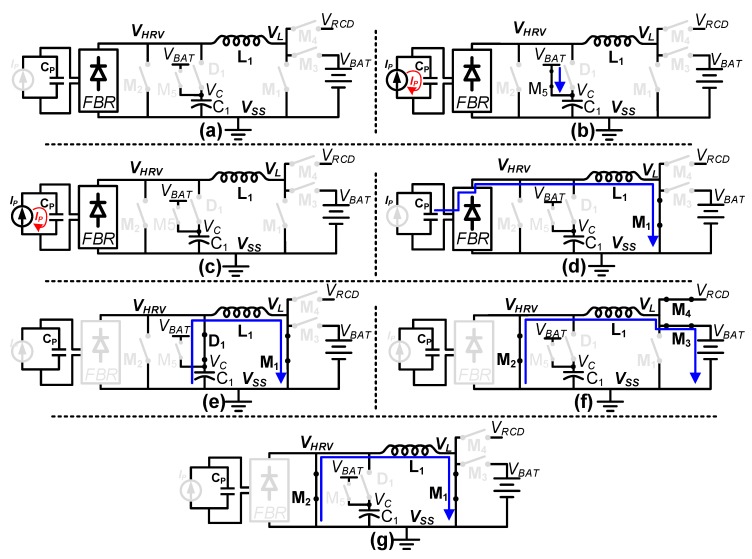
Proposed harvesting interface operation states (**a**) Standby; (**b**) Precharge; (**c**) Voltage peak detection; (**d**) Inductor charging; (**e**) Free-wheeling; (**f**) Battery charging; (**g**) Reset.

The operation of the proposed harvesting interface involves seven different states, as shown in Figure 3. In each state, the connected (closed) switches are shown in black, whereas the disconnected (open) switches are shown in gray. When the harvester output voltage *V_HRV_*, which is input for the harvesting interface, is less than 5 V, the harvesting interface is in standby state, and all switches are left open as shown in Figure 3a. In this state, only the WUC is active, continuously monitoring *V_HRV_* for harvesting activation. As *V_HRV_* exceeds 5 V with motion applied to the harvester, the WUC generates a wake up request signal (*WU_OUT_* in Figure 2) to activate the harvesting process. Upon *WU_OUT_* assertion, the WUC is power-gated and the VPD is enabled. At the same time, the storage capacitor C_1_ is charged to V_BAT_ through M_5_ with 1 µs precharge pulse, as shown in Figure 3b. After the precharge, all of the switches are opened again as shown in Figure 3c until the *V_HRV_* peak is detected by the VPD when the motion applied to the harvester ceases. As the peak is detected, M_1_ is turned on to transfer energy from the harvester’s internal capacitor (C_P_) to an inductor (L_1_) as shown in Figure 3d.

While L_1_ is being energized, the VPD is power-gated and the IPD is enabled to detect when the inductor current is peaking. As the energy is transferred from C_P_ to L_1_, *V_HRV_* drops below the precharged voltage of C_1_, i.e., *V_BAT_*, and D_1_ turns on as shown in Figure 3e. The IPD detects when *V_HRV_* drops below *V_SS_* and then triggers *IPD_OUT_* in Figure 2 to power-gate the IPD and enable the RCD. One thing to note is that the IPD is implemented with a low-voltage (LV = 5 V) device available in the HV/LV BCD process. Therefore, the IPD cannot directly compare *V_HRV_* with *V_SS_* to detect the inductor current peak since *V_HRV_* can exceed the LV device breakdown limit during energy harvesting. Therefore, instead of using *V_HRV_*, diode-protected voltage *V_C_* is compared with *V_SS_*. For proper operation, an initial positive potential is required at *V_C_*, which is provided by LV PMOS (M_5_) and a capacitor (C_1_) as described in the precharge phase shown in Figure 3b.

**Figure 4:**
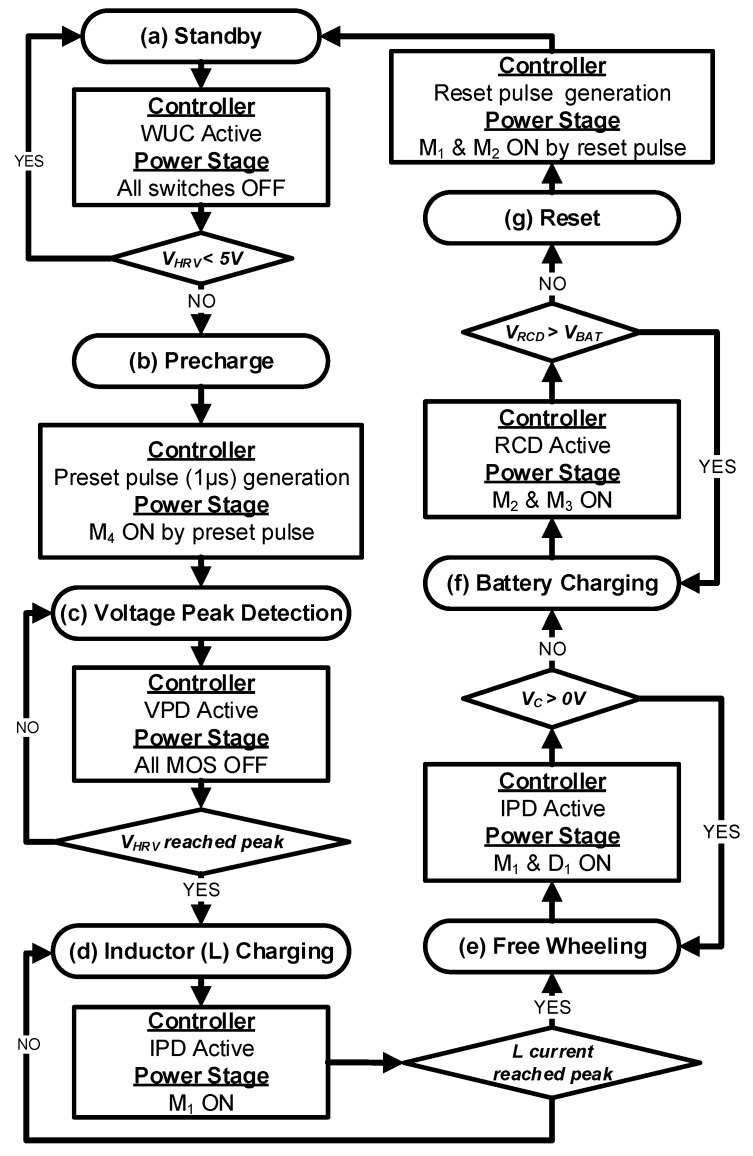
Proposed harvesting interface operation flow chart.

As the inductor current peak is detected, energy is transferred from L_1_ to the battery by connecting M_2_/M_3_ as shown in Figure 3f. As the energy is transferred to the battery, the RCD monitors *V_RCD_* and *V_BAT_* so that reverse current can be detected when *V_L_* falls below *V_BAT_*. One thing to note here is that the RCD also has been implemented with LV devices as shown in Figure S1; hence, *V_L_* cannot be directly connected to RCD as it can exceed the LV device breakdown limit when M_3_ is turned off. To address this issue, a gated version of *V_L_*, named *V_RCD_,* is connected to the RCD instead of *V_L_*. *V_L_* and *V_RCD_* are connected through an HV NMOS M_4_, which is turned on when the RCD is enabled, and M_3_ is turned on for energy transfer to the battery. Therefore, *V_RCD_* can remain within the LV device breakdown limit. *V_RCD_* is higher than *V_BAT_* as long as current flows from the inductor to the battery. At complete energy transfer from the inductor to the battery, reverse current starts to flow from the battery, resulting in lower *V_RCD_* as compared to *V_BAT_*, indicating reverse current. At reverse current detection, transistors M_3_/M_4_ are opened and a reset pulse *RST* (in Figure 2) is generated. During *RST*, L_1_ residual energy is dissipated by shorting the inductor terminals to *V_SS_* through M_1_ and M_2_ as shown in Figure 3g. At the falling edge of *RST*, an energy harvesting cycle is completed, and the harvesting interface returns to standby state where all of the switches are open and only the WUC remains activated. The operational flow chart of the proposed harvesting interface is shown in Figure 4, which illustrates the behavior of the controller and power stage in each state shown in Figure 3. 

**Figure 5:**
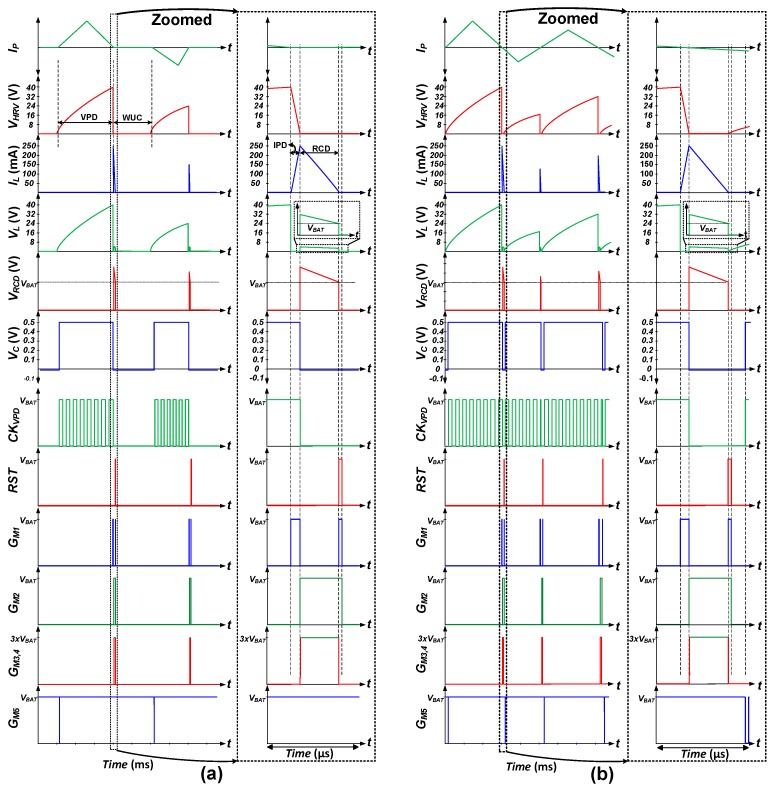
Proposed harvesting interface conceptual timing waveforms at (**a**) Discontinuous input harvesting and (**b**) Continuous input harvesting.

Figure 5 shows the conceptual waveform of the proposed harvesting interface operation with harvesting from discontinuous (Figure 5a) and continuous (Figure 5b) input (*V_HRV_*). This figure shows that the energy transfer from C_P_ to battery requires only a few microseconds (µs). Since the typical motions that kinetic energy harvesting is based on are on the order of milliseconds (ms) or seconds, e.g., human motion or machine vibration, this µs order power transfer scheme allows energy harvesting under discontinuous as well as continuous excitation conditions.

## 3. Controller and Driver Implementation Details

### 3.1. Wake-Up Controller (WUC)

Harvesting under sporadic excitation introduces long standby periods between successive energy inputs. If the entire harvesting interface is powered on during this period, a significant amount of energy can be consumed while waiting for the next energy input. Therefore, to minimize energy consumption between harvesting inputs, minimal monitoring operation is activated, and the rest of the circuit is powered off. For this reason, the WUC is designed to monitor energy input with minimal power consumption and trigger harvesting operation whenever there is energy input, as described earlier and shown in Figure 4 and Figure 5.

**Figure 6:**
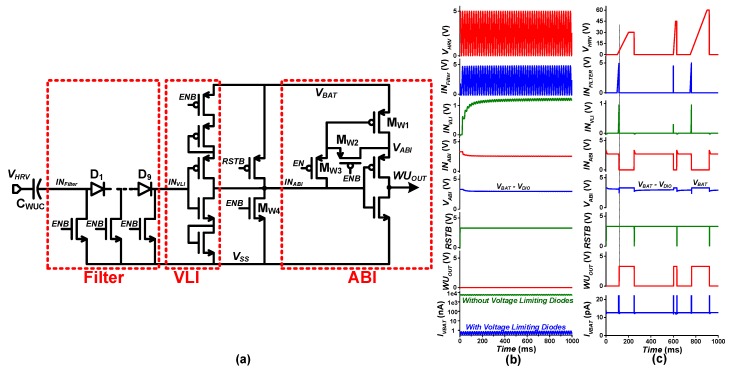
Proposed low quiescent current wake-up controller (WUC): (**a**) Circuit, (**b**) Non-harvestable low voltage input waveforms, (**c**) Normal operation waveforms.

The transistor-level design of the proposed WUC circuit, presented in Figure 6a, consists of a low voltage noise filter, a voltage-limiting inverter (VLI), and an adaptable biasing inverter (ABI). The simulated waveforms are shown in Figure 6b,c.

With weak mechanical excitations, the harvesting source, such as PZT or TENG, generates low voltage pulses as shown in Figure 6b, with harvestable energy as low as a few nanoJoules per excitation. As the harvesting interface’s activation energy overhead is on the order of ~10 nJ, activating harvesting operation for such weak input wastes energy. To prevent harvesting activation in such conditions, a low energy input filter is adopted at the first stage of the WUC, which is followed by a diode voltage limiting inverter (VLI). Figure 6b shows that at a non-harvestable voltage limit of *V_HRV_* = 5 V, the VLI limits the short circuit (SC) current through the WUC to less than 1 nA, which ranges up to a few µA without voltage-limiting diodes as shown in Figure 6b. As shown in Figure 6a, the harvester output (*V_HRV_*) is capacitively coupled through an external capacitor C_WUC_ to avoid IC damage due to high input voltage. Capacitively coupled input voltage (*IN_Filter_*) is filtered through a stack of nine diodes with *V_DIO_* = 0.4 V. Therefore, the following VLI is not triggered until *IN_Filter_* = 0.5 × *V_BAT_* + 9 × 0.4 ≈ 5 V, at which point *IN_VLI_* becomes 0.5 × *V_BAT_*, allowing filtering of low input voltages.

When *V_HRV_* (and *IN_Filter_*) < 5 V, the VLI input (*IN_VLI_*) remains lower than VLI’s switching threshold voltage (*V_M_*) and provides high output (*V_BAT_* − *V_thp_*) at node *IN_ABI_*, the input to the ABI. To prevent short circuit current through the ABI due to threshold voltage drop (*V_thp_*) at *IN_ABI_*, the supply voltage of the ABI is set to *V_ABI_* = *V_BAT_* − *V_thp_* by using M_W1_ with diode configuration, i.e., shorting its gate and source terminals through M_W2_.

**Figure 7:**
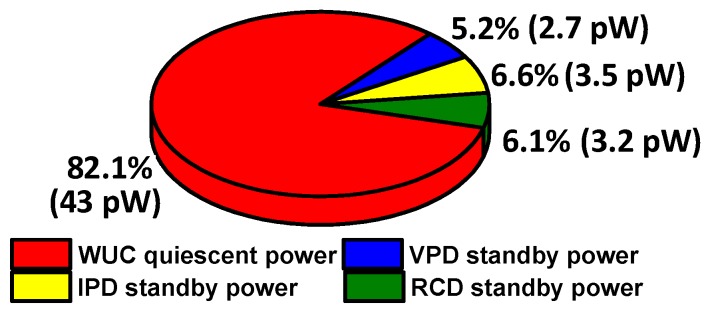
Harvesting interface standby mode power consumption pie chart.

When the input rises to *V_HRV_* (and *IN_Filter_*) ≥ 5 V, *IN_ABI_* falls to *V_thn_*, where *V_thn_* is the threshold voltage of limiting NMOS in the VLI, resulting in high transition at WUC output (*WU_OUT_* = *V_BAT_* − *V_thp_*). Upon such transition, the WUC activation control signal *(EN/ENB*) is disabled (*EN* = 0 V*, ENB = V_BAT_*), and *IN_ABI_* is pulled down to *V_SS_* by M_W4,_ whereas *V_ABI_* is pulled up to *V_BAT_* by turning OFF/ON M_W2_/M_W3_, respectively. Thus full swing wake-up output (*WU_OUT_* = *V_BAT_*) is generated. As *WU_OUT_* is asserted, the WUC is disabled until a reset pulse (*RST*) is triggered to enable the WUC (*EN* = *V_BAT_, ENB =* 0 V) at the end of the harvesting operation cycle as described in Section 2. The proposed WUC has been implemented with all LV devices except a 10 pF high-voltage coupling capacitor (C_WUC_ = 06035A100JAT2A). To avoid high voltage stress at the WUC’s LV devices, the C_WUC_ bottom plate is connected to *V_SS_* while WUC is disabled during harvesting operation. Voltage limitation at the VLI and adaptive biasing at the ABI result in ultra-low WUC quiescent current on the order of a few pico-amperes (at *V_HRV_* = 0 V) to 100s of pA (at *V_HRV_* = 5 V). As described earlier, since only the WUC is active during standby mode, standby power consumption of the harvesting interface is dominated by the WUC, making the entire harvesting interface’s standby power only tens of pW as illustrated in Figure 7, which is lower as compared to the prior arts [12,13,14].

### 3.2. Voltage Peak Detector (VPD)

As described in Figure 3, once the harvesting interface is awoken from the standby state (in Figure 3) by the WUC, the storage capacitor (C_1_) is precharged, and no further action is taken until the peak of input is detected. A VPD circuit is proposed to detect such an input peaking event by monitoring the slope of *V_HRV_*. Under application of uni-directional mechanical excitation, *V_HRV_* increases and the slope of *V_HRV_* remains positive. When mechanical excitation is stopped (pulsed excitation) or the direction is reversed (periodic excitation), the slope of *V_HRV_* becomes negative due to leakage current or *I_P_* polarity reversal, and such slope polarity change can be sensed for detecting a *V_HRV_* peaking event.

Figure 5 shows that VPD activation dominates the harvesting interface active mode period as it remains activated for a few ms, whereas IPD and RCD are activated only for a small fraction (a few µs) of the harvesting interface active mode duration. Thus, to minimize the control power overhead during the harvester interface active mode, an efficient VPD is critical.

**Figure 8:**
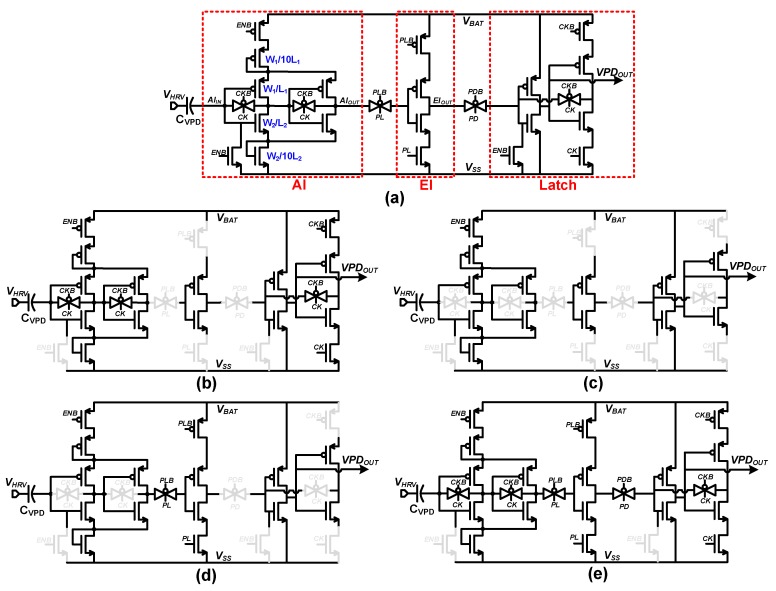
Proposed voltage peak detector (VPD): (**a**) Circuit, (**b**) Reset phase, (**c**) Amplification phase, (**d**) Evaluation phase, and (**e**) Latch update phase.

**Figure 9:**
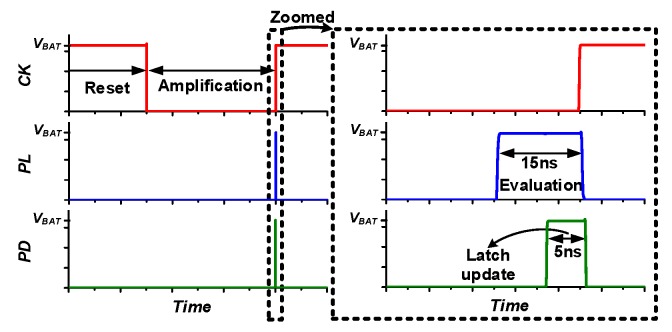
Voltage peak detector timing waveform.

Figure 8 shows the details of the proposed VPD, which consists of amplification inverters (AI), an evaluation inverter (EI), and a latch. In the proposed design, voltage-limiting and current-starving schemes are implemented by diode-connected long header and footer PMOS/NMOS, limiting current consumption though the AI. In contrast, a limited on-time approach is used to limit EI power consumption. The proposed VPD operates with four phases: Reset, Amplification, Evaluation and Latch Update. The operation details of each phase are illustrated in Figure 8b–e, and the corresponding timing waveforms are shown in Figure 9.

In the Reset phase, the clock (*CK*) is set high, and VPD is reset by enabling input-output shorting transmission gates of the AI. Thus, AI input and output are biased to its switching threshold (*V_M_*) voltage. Meanwhile, the EI is disabled, and latch holds the previously stored value as shown in Figure 8b (Figure 9 “Reset” period). As the *CK* is toggled, the VPD enters the Amplification phase, and the AI reset is released and acts as an inverting amplifier with high gain. During this phase, the change in *V_HRV_*, which is coupled through *C_VPD_*, is amplified through an inverter chain, whereas EI remains disabled, and the latch feedback path is opened as shown in Figure 8c (Figure 9 “Amplification” Period). Before the *CK* is restored to high, a ~15 ns pulse (*PL*) is generated to start the Evaluation phase. In this phase, the EI is enabled as shown in Figure 8d (Figure 9 “Evaluation” period), and the amplified slope signal generates a digital signal (0 V*, V_BAT_*) at *EI_OUT_* to indicate the rise or fall transition at *V_HRV_*. *EI_OUT_* is transferred to output latch by a second ~5 ns pulse (*PD*), which forms the Latch Update phase as shown in Figure 8e (Figure 9 “Latch update” period), and the output is fully latched by raising the *CK* and pulling down *PL* and *PD*, which make the VPD start over from the Reset phase. For stable operation of the latch, there is ~2 ns overlap time after *CK* rises and *PL*/*PD* falls. Since the slope is detected once every cycle, the *CK* frequency needs to be adjusted according to the characteristics of harvesting input. To provide efficient operation over a wide range of input with varying period, the *CK* frequency is made tunable from 10 Hz to 1 kHz. For VPD clock (*CK*) generation, a leakage control clock generator [21], which provides frequency tunability by controlling leakage control voltage (*V_CONT_*), and consumes 110 pJ/*CK*_cycle have been adopted. The details of the adopted *CK* generator have been shown in supplementary Figure S2. The clock generator of the proposed VPD provides 200 Hz clock signal (*CK*) at the default setting, which is high enough for harvesting kinetic energy from, for example, human motion.

**Figure 10:**
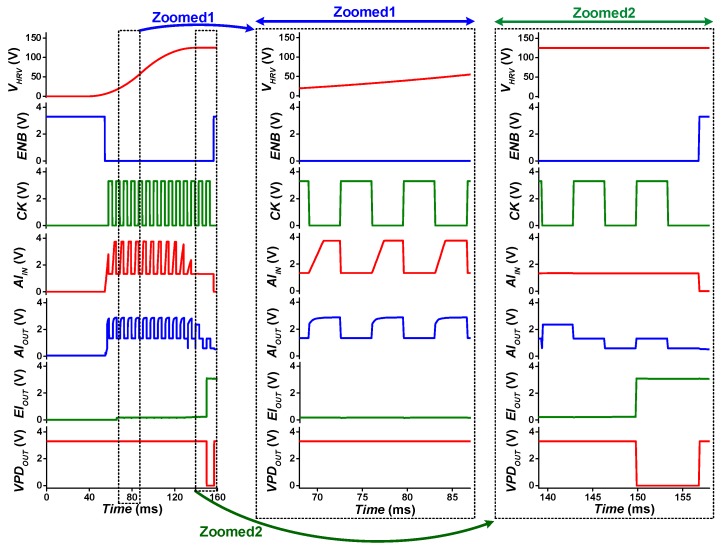
Voltage peak detector operation waveform.

The operation of the proposed VPD is illustrated with simulation waveform in Figure 10. When kinetic energy is converted to electrical energy through the harvesting source, the slope of *V_HRV_* remains positive, which keeps VPD output (*VPD_OUT_*) = *V_BAT_* (Figure 10 Zoomed1). When *V_HRV_* reaches the peak voltage as no more kinetic energy is harvested, the *V_HRV_* slope becomes negative due to the parasitic leakage current of the harvesting interface and harvesting source. Such change in *V_HRV_* slope toggles latch output, and *VPD_OUT_* triggers low (Figure 10 Zoomed2), indicating a *V_HRV_* peaking event.

Current starving at the AI and the limited activation time of EI result in nanoamperes VPD active mode current consumption. High slope sensitivity and nanoamperes current consumption of the proposed VPD limit the controller power overhead.

The proposed VPD is implemented with all LV devices except an external 10 pF high-voltage coupling capacitor (C_VPD_ = 06035A100JAT2A). Therefore, protection of the LV devices is required just like the WUC. In the proposed VPD, if *V_HRV_* increases by more than (*V_BAT_ + V_TH_*) in a single clock cycle, the potential at the bottom plate of the coupling capacitor is limited to (*V_BAT_ + V_TH_*), as shown in Figure 10 (Zoomed1), through the PMOS of the reset transmission gate and NMOS of the first amplification inverter. Thus, the proposed VPD inherits HV protection without any additional architecture or power overhead.

### 3.3. Two-Stage Bootstrap Driver

**Figure 11:**
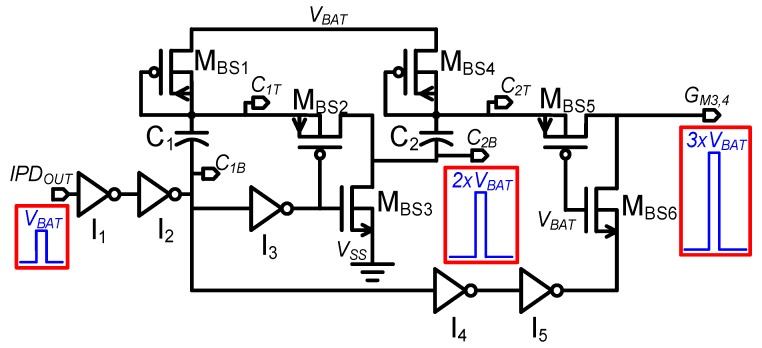
Two-stage bootstrap driver.

In the proposed harvesting interface shown in Figure 2, the source terminals of HV NMOS M_1_, M_2_ and LV PMOS M_5_ are connected to *V_SS_* and *V_BAT_*, respectively. Thus, gate terminals can be driven by signals with voltage swing of *V_SS_* to *V_BAT_*. However, the source terminals of HV NMOS M_3_ and M_4_ are connected to *V_BAT_* and hence require a signal with a voltage level higher than *V_BAT_* to turn them on. Higher driving voltage for turning on M_3_ and M_4_ is better for smaller conduction loss. To control M_3_ and M_4_ with signal with voltage swing from 0 V to ~3 × *V_BAT_*, a two-stage bootstrap driver is implemented as shown in Figure 11 with LV devices for minimized switching loss within the bootstrap driver.

**Figure 12:**
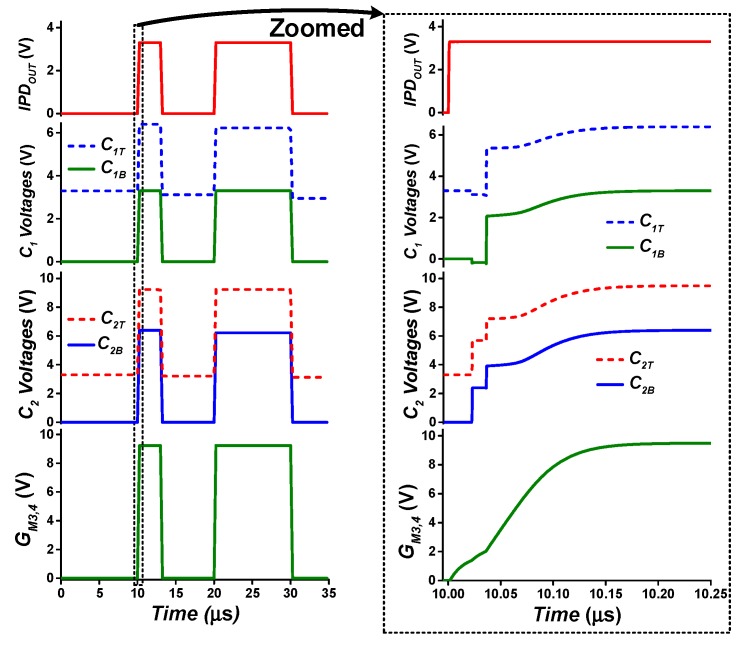
Two-stage bootstrap driver operation waveform.

The operation of the proposed bootstrap driver with simulated waveforms is shown in Figure 12. At low input (*IPD_OUT_* = 0 V), the top plates of capacitors C_1_ and C_2_ (*C_1T,_ C_2T_* ) are at (*V_BAT_* − *V_thp_*) potential due to the threshold voltage drop of the diode-connected transistors M_BS1_ and M_BS4_, respectively. On the other side, the capacitor bottom plates (*C_1B_, C_2B_*) are at *V_SS_* through inverter I_2_ and transistor M_BS3_, respectively. The gate terminals of transistors M_BS5_ and M_BS6_ are permanently tied to *V_BAT_*, enabling M_BS5_ and disabling M_BS6_ at low *IPD_OUT_*. As a result, driver output (*G_M3, 4_*) is set to low (0 V).

Upon low-to-high input transition (*IPD_OUT_* = *V_BAT_*), *C_1B_* is pulled up to *V_BAT_*. Due to capacitive coupling, *C_1T_* potential increases to 2 × *V_BAT_* – *V_thp_*, and M_BS1_ is turned off with reverse bias, preventing reverse current conduction from *C_1T_* to *V_BAT_*. Inverter I_3_ pulls down the gates of M_2_ and M_3_ to *V_SS_*, which turns on/off M_2_/M_3,_ respectively. Thus, the *C_2B_* node potential changes from 0 V to 2 × *V_BAT_* − *V_thp_*, which then increases the potential at *C_2T_* from *V_BAT_* to 3 × *V_BAT_* − 2 × *V_thp_* by capacitor coupling. As the gates of M_BS5_ and M_BS6_ are tied to *V_BAT_*, and the gate/body of M_BS4_ are tied to *C_2T_*, high transition at *C_2T_* turns off/on M_BS4_/M_BS5_, respectively. Meanwhile, M_BS6_ is turned off by connecting its source terminal to *V_BAT_* potential through inverter I_5_. Thus, the gate driving signal *G_M3, 4_* transitions from 0 V to 3 × *V_BAT_* − 2 × *V_thp_*.

In the proposed driver topology, the maximum potential difference across all devices remains within 2 × *V_BAT_*, which complies with the potential limit of low-voltage devices. Thus, the proposed bootstrap driver architecture improves efficiency by lowering switching loss because it generates approximately 3 × *V_BAT_* voltage swing drive signal by using only low-voltage devices.

## 4. Simulation Results

**Figure 13:**
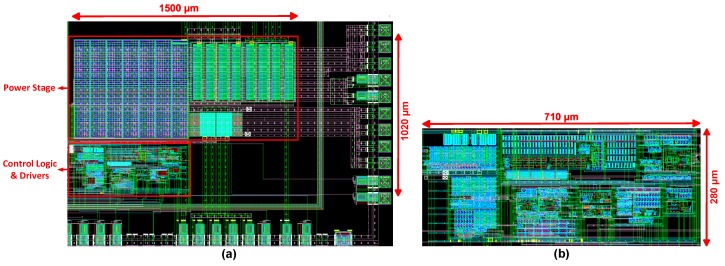
Layout of the proposed harvesting interface with (**a**) integrated power stage version and (**b**) discrete devices power stage version.

The proposed random input harvesting interface is designed in 350 nm HV/LV (40 V/5 V) BCD process and evaluated with post-layout simulation with Hspice and Spectre. The BCD process used for circuit design provides high and low drain-source voltage devices. High-voltage devices offer ratings up to 40 V (break-down rating ≈ 48 V), and low-voltage devices have a rating of 5 V (break down rating ≈ 8 V). By utilizing both types of devices, two versions of the proposed harvesting interface are designed. In the first version, all high-voltage switches in the power stages are implemented with 40 V-rated integrated devices. Figure 13a shows the layout of the proposed harvesting interface with all power switches integrated. The power stage occupies an active area of 1500 µm × 660 µm, whereas the controller and drivers occupy 712 µm × 280 µm. For the second version, 200 V-rated discrete devices are assumed to be used as power switches, and only the controller and drivers are implemented on-chip with low-voltage devices as shown in Figure 13b. Due to the low voltage rating of the integrated capacitor (5 V) provided by the selected technology, discrete capacitors are used for all high-voltage capacitors. For simulating both versions, a 220 µH inductor with internal resistance (R_DC_) of 0.022 Ω (Model = SRR7032) is used.

To verify operation of the proposed harvesting interface, two different piezoelectric generator (PEG) models are examined. The first model is a custom-made flexible PEG with low internal capacitance used in [19] whose internal capacitance (C_P_) is 500 pF and internal resistance (R_P_) is 2 GΩ. The second model is a commercial PEG “MIDE V22B” with an internal capacitance of 19.5 nF.

For simulations, a PEG is often modeled with a variable current source, an internal capacitance and a resistance as shown in Figure S3a [4,5,6,11,12,13,14]. A variable current source is used to model various input conditions; it is assumed that the current source generates triangular current pulse with fixed duration (100 ms) and varying amplitude to model varying forces applied to the PEG. Such variation in physical input conditions results in variation in the generated energy (*E_HRV_IN_*) and the resulting peak open circuit voltage (*V_HRV(OC)_PEAK_*). *V_HRV(OC)_PEAK_* is the voltage at the end of physical deformation of the harvesting material when no additional load is attached to it (Figure S3b). As the amplitude of the current source (*I_P_*) varies, *V_HRV(OC)_PEAK_* also varies from 10 V to 195 V, as shown in Figure S3c. For the rest of measurement, the input condition is represented with this *V_HRV(OC)_PEAK_* since the actual peak voltage during harvesting operation varies with the input capacitance of the attached load circuits—the harvesting interface or a full bridge rectifier (FBR).

**Figure 14:**
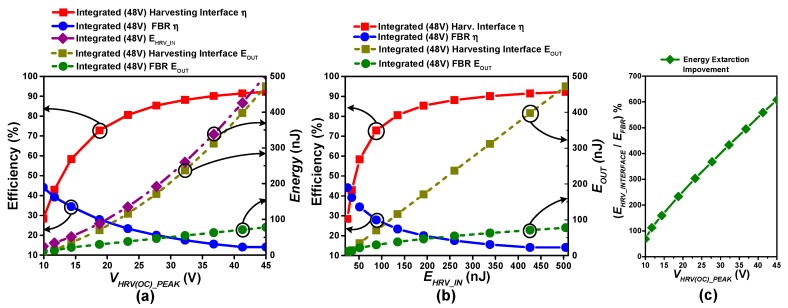
(**a**,**b**) Efficiency comparison between the FBR and the proposed harvesting interface with integrated power switches at harvester open circuit voltage and input energy on x-axis respectively when harvesting from a flexible piezoelectric harvester source (C_P_ = 500 pF) under irregular excitation at 3.3 V battery charging, (**c**) Energy extraction improvement w.r.t FBR.

Figure 14 compares the harvesting performance of the integrated version of the proposed harvesting interface and an FBR. Post-layout simulation is conducted to measure the efficiency (left Y-axis) and harvested output energy (*E_OUT_*) (right Y-axis) for charging a 3.3 V battery. Figure 14a shows them as functions of *V_HRV(OC)_PEAK_* (abbreviated as *V_HRV(OC)_* in figures), and Figure 14b shows them as functions of *E_HRV_IN_*. As *V_HRV(OC)_PEAK_* is increased with stronger input excitation, more energy is generated by the PEG. When the proposed harvesting interface is utilized, the harvesting operation is not activated until the output voltage reaches its peak, i.e., excitation is finished, allowing voltage accumulation at the output of the PEG. The final voltage when the harvesting interface is connected, *V_HRV(INT)_PEAK_,* is smaller than *V_HRV(OC)_PEAK_* due to larger load capacitor seen by the harvesting source since the input capacitance of the harvesting interface is added. Assuming the total charge generated by the PZT harvester is fixed with identical excitation, the relation between *V_HRV(OC)_PEAK_* and *V_HRV(INT)_PEAK_* can be expressed as:

(3) $$Q=C_{P}\cdot V_{HRV\left(OC\right)\_PEAK\;}=\left(C_{P}+C_{IN}\right)\cdot V_{HRV\left(INT\right)\_PEAK}$$

where C_P_ is the parasitic capacitance of the PEG, and *C_IN_* is the input capacitance of the proposed harvesting interface. Since the amount of energy generated by the PEG can be calculated as:

(4) $$E=\frac{1}{2}CV^{2}$$

The PEG-generated energy (*E_HRV_IN_*) is proportional to ${\left(V_{HRV\left(INT\right)\_PEAK}\right)}^{2}$ and also to ${\left(V_{HRV\left(OC\right)\_PEAK}\right)}^{2}$. Therefore, the overall energy harvested by the harvesting interface (*E_OUT_*) is also approximately proportional to ${\left(V_{HRV\left(OC\right)\_PEAK}\right)}^{2}$, as shown in Figure 14a, and approximately proportional to *E_HRV_IN_*, as shown in Figure 14b. On the other hand, when an FBR is used for harvesting, the output voltage of the PEG is limited to *V_FBR_HARV_* = *V_th_FBR_ + V_BAT_*, where *V_th_FBR_* is the FBR threshold voltage (Figure S3d,e). Therefore the amount of energy generated by the PEG is proportional to the amount of charge generated by the PEG, making the amount of harvested energy with an FBR (*E_OUT_*) also approximately proportional to $V_{HRV\left(OC\right)\_PEAK\;}$, as shown in Figure 14a.

In this measurement, the efficiency of the proposed harvesting interface or FBR is defined as follows:

$$\mathrm{Efficiency}=\frac{\mathrm{Amount}\;\mathrm{of}\;\mathrm{energy}\;\mathrm{harvested}\;\mathrm{with}\;\mathrm{harvesting}\;\mathrm{circuit}-\mathrm{Operation}\;\mathrm{overhead}}{\mathrm{Amount}\;\mathrm{of}\;\mathrm{energy}\;\mathrm{generated}\;\mathrm{by}\;\mathrm{PEG}\;\mathrm{in}\;\mathrm{open}\;\mathrm{circuit}\;\mathrm{condition}\;\left(E_{\mathrm{HRV}\_\mathrm{IN}}\right)}$$

With a weak excitation on the PEG, i.e., low *V_HRV(OC)_PEAK_* or *E_HRV_IN_*, the efficiency of the proposed harvesting interface drops due to the circuit operation overhead, resulting in worse efficiency than that achieved with an FBR. But as *V_HRV(OC)___PEAK_* exceeds 11.5 V (or *E_HRV_IN_* exceeds 33 nJ), the efficiency of the harvesting interface exceeds that of the FBR, with a maximum energy extraction improvement of up to 607% at *E_HRV_IN_* = 506 nJ *(V_HRV(OC)_PEAK_ =* 45 V). Detailed simulated waveforms of the proposed harvesting interface at this point are provided in supplementary Figure S4. Figure 14c shows the percentage improvement in energy extraction of the proposed interface as compared to an FBR. It can be clearly seen that a significantly larger amount of energy can be harvested with the proposed harvesting interface than with an FBR.

**Figure 15:**
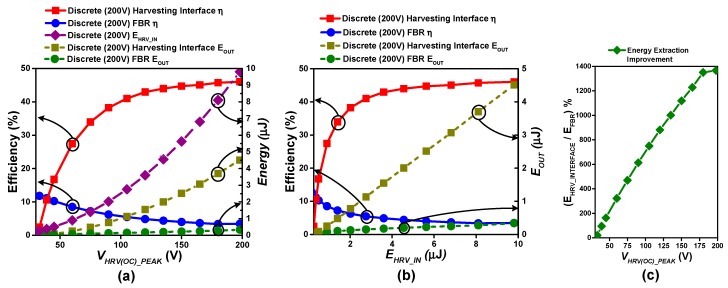
(**a**,**b**) Efficiency comparison between the FBR and the discrete power switches version of the proposed harvesting interface at harvester open circuit voltage and input energy on x-axis respectively when harvesting from flexible piezoelectric harvester source (C_P_ = 500 pF) under irregular excitation, at 3.3 V battery charging, (**c**) Energy extraction improvement w.r.t FBR.

As mentioned earlier, the 350-nm BCD process utilized for performing post-layout simulation provides 40 V high-voltage devices whose break down rating is 48 V. Therefore, to harvest energy with input peak voltage *V_HRV(INT)_PEAK_* higher than 48 V, integrated power switches cannot be used as is. A version of the proposed harvesting interface is designed to operate with discrete high voltage transistors with 200 V rating (HV NMOS = PHC2300, FBR = DF02M, Diode = BAS21). Figure 15 shows the simulation results for efficiency and *E_OUT_* with the discrete power switches and with an FBR. Similar to the earlier case, a flexible PEG model is used, and the proposed harvesting interface is compared with a discrete FBR for charging a 3.3 V battery. Spice simulation models from vendors of discrete devices are utilized for analysis. Discrete high-voltage devices inherit higher parasitic loading, leakage loss, and driving overhead as compared to integrated versions; thus, in general, the efficiency is lower than that of the integrated version. FBR performance is better than that of the discrete version of the proposed interface up to *V_HRV(OC)_PEAK_ =* 40 V. But, as stronger excitation is applied to the PEG and *V_HRV(OC)_PEAK_* exceeds 40 V (*E_HRV_IN_* > 400 nJ), the efficiency of the discrete HV device harvesting interface exceeds that of the FBR. Figure 15c shows that the proposed topology provides up to 1365% efficiency improvement as compared to the FBR when *E_HRV_IN_* = 9.8 µJ *(V_HRV(OC)_PEAK_* = 195 V). The overall trends of efficiency and *E_OUT_* are similar to the results obtained with the integrated version.

**Figure 16:**
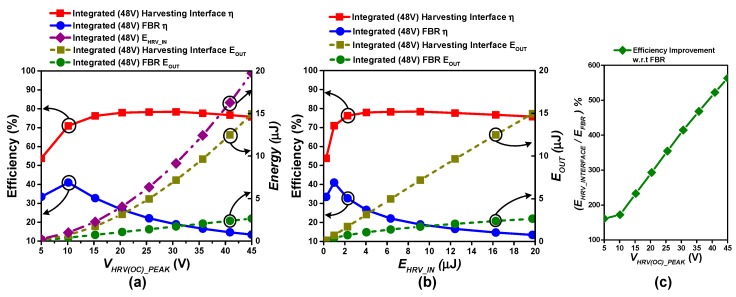
(**a**,**b**) Efficiency comparison between the FBR and the proposed harvesting interface with integrated power switches at harvester open circuit voltage and input energy on x-axis respectively when harvesting from MIDE V22B piezoelectric harvester source (C_P_ = 19.5 nF) under periodic excitation at 3.3 V battery charging, (**c**) Energy extraction improvement w.r.t FBR.

Unlike the custom-built flexible PEG [19], typical commercially available PEGs have internal capacitances on the order of ten nF_._ To evaluate the compatibility of the proposed interface with commercial PEGs, the proposed harvesting interface was simulated with a PEG model whose internal capacitance is similar to “MIDE V22B,” whose *C_P_* is 19.5 nF. Figure 16a,b show the post-layout simulation results for efficiency and *E_OUT_* with the integrated version of the proposed harvesting interface and an FBR. 

The simulations are performed with varying levels of sinusoidal excitations, and the frequency of the excitation is assumed to be 174 Hz, which is the resonance frequency of MIDE V22B. The overall trends of efficiency and *E_OUT_* are similar to the earlier analysis. However, due to the high energy-generation capacity and resulting capacitance of the harvester source, harvestable energy even at the low voltage limit (*V_HRV(OC)_PEAK_* = 5 V) is significantly higher than that of the control and driving overhead, making the proposed harvesting interface superior than the FBR for all input ranges. Figure 16c shows that the proposed topology provides up to 562% efficiency improvement as compared to the FBR when *V_HRV(OC)_PEAK_ =* 45 V (*E_HRV_IN_ =* 19.7 µJ) (for energy to power conversion multiply energy results with double of the periodic excitation frequency (348), because a positive and negative pulse is generated during each excitation). The detailed waveforms obtained under this condition are provided in supplementary Figure S5. As the high capacitance PEG MIDE V22B can provide a maximum *V_HRV(OC)_PEAK_* of ~40 V in real measurement, the discrete version of the proposed interface has not been simulated with the high capacitance PEG model.

A performance comparison of the proposed harvesting interface with other prior arts [11,12,13,14] is shown in Table 1. The proposed harvesting interface is designed to handle a wide range of input voltages for maximum energy extraction. The maximum input voltage can be easily extended to 200 V with the use of discrete devices since the all-NMOS power stage design only requires control signals as high as 2 × *V_BAT_* or 3 × *V_BAT_* for handling high voltage inputs. The proposed harvesting interface can handle irregular pulsed inputs as well as periodic inputs with a load isolation scheme. Low operation overhead is achieved with event-based activation of an all-NMOS power stage and energy efficient WUC, VPD implementations, resulting in a large energy extraction improvement up to 1365%. Event-based activation with a low quiescent power WUC allows extremely low standby power consumption of 53 pW, enabling efficient energy harvesting from sporadic energy inputs with long intervals.

## 5. Conclusions

A novel harvesting interface has been introduced in this work for harvesting kinetic energy under irregular as well as periodic excitation. The proposed harvesting interface operates with a load isolation scheme to maximize energy extraction, which allows efficient harvesting independent of the nature of the harvester source or the type of excitation. Thus, the proposed interface operates efficiently over a wide voltage range and can be attached to a variety of harvester sources, from a PEG to a TEG, and under irregular as well as periodic excitation. A new WUC with ~13 pA quiescent current has been presented in this work, significantly reducing standby power and making the proposed harvesting interface suitable for energy harvesting from sporadic inputs. With an all-MOS power stage and an energy efficient VPD, efficient harvesting operation is enabled to significantly improve energy extraction.

The post layout analysis verifies that the integrated version of the proposed harvesting interface provides higher efficiency compared with that of an FBR over the *V_HRV(OC)_* range of 11.5 V to 45 V, with a peak efficiency improvement of 607% at 45 V when a PEG with a small C_P_ of 500 pF is used as the source. When harvesting from the PEG with a higher C_P_, such as the commercially available PEG “MIDE V22B,” whose C_P_ is 19.5 nF, the proposed interface efficiency is always higher than that of the FBR, with a peak improvement of 562% at 45 V. The proposed harvesting interface can also operate with discrete power switches, and our analysis with discrete NMOS with a voltage rating of 200 V provides high efficiency as compared to an FBR over a *V_HRV_* range of 40 V to 195 V with peak efficiency improvement of 1365% at 195 V when a PEG with C_P_ of 500 pF is used as the harvester source.

We believe that the proposed harvesting interface can be used for efficient harvesting under random excitation, enabling efficient energy harvesting in numerous new applications, such as charging portable electronics with energy generated from random human body motion and charging wireless sensor nodes attached to animals for tracking by harvesting energy from random animal motions.

## Figures and Tables

**Table 1:** Performance comparison with prior harvesting interfaces.

Parameters	This Work	[11]	[12]	[13]	[14]
**Technology**	350 nm BCD	250 nm HV	350 nm HV	40 nm HV	350 nm HV
**Operation Principle**	Load Isolation	Bias flip + SC Series/Parallel	PSCE	SECE	Energy Investing
***V_HRV_*** **(V)**	5–45 (INT*) 5–200 (DIS*)	<35	<20	<6	<6
***V_OUT_* (V)**	2.4–4	2.5	1.5–5	1.5, 2.8	3.3
**Harvester Capacitance**	500 pF, 19.5 nF	150 nF	19.5 nF	43 nF	15 nF
**Off-chip Inductor**	220 µH	470 µH	2.2 mH	2.2 mH	330 µH
**Excitation Type**	Irregular & Periodic	Irregular	Periodic	Periodic & Shock	Periodic & Shock
**Efficiency Improvement** **(w.r.t FBR)**	607% (INT) 1365% (DIS)	-	206% (Periodic)	420% (Shock) 314% (Periodic)	360% (Periodic)
**Harvesting Overhead** **(Interface Active Mode Power)**	1.6 µW (INT) 2.6 µW (DIS) @ 173 Hz (Continuous *V_HRV_*)	12 µJ (Per pulse)	4.4 µW @ 173 Hz (Continuous *V_HRV_*)	-	1.6 µW @ 143Hz (Continuous *V_HRV_*)
**Quiescent Current/Power** **(Interface Standby Mode Power)**	16 pA/53 pW @*V_BAT_ =* 3.3 V	-	300 nA	30 nA/45 nW	100 nA/320 nW

INT* = version with integrated power stage devices; DIS* = version with discrete power stage devices.

## Supplementary Materials

The following are available online at https://www.mdpi.com/1424-8220/19/17/3685/s1: Figure S1, Current peak detector (IPD) and reverse current detector (RCD) circuits; Figure S2, Leakage control clock generator; Figure S3, Harvester source model, open circuit peak voltage variation by varying fixed duration (100 ms) harvester’s triangular current pulse amplitude and FBR harvesting; Figure S4, Proposed harvesting interface operation at discontinuous harvesting from flexible piezoelectric harvester (C_P_ *=* 500 pF) at *V_HRV(OC)_PEAK_* = 45 V; Figure S5, Proposed harvesting interface operation at periodic harvesting at 173 Hz from MIDE V22B harvester (C_P_ = 19.5 nF) at *V_HRV(OC)_PEAK_* = 45 V.
