# Supplementary material for: A High-Voltage Energy-Harvesting Interface for Irregular Kinetic Energy Harvesting in IoT Systems with 1365% Improvement Using All-NMOS Power Switches and Ultra-low Quiescent Current Controller
Source: Sensors (Basel). 2019 Aug 24;19(17):3685. doi: 10.3390/s19173685 (PMC6749379; doi:10.3390/s19173685)
Supplement: Supplementary file 1 [file sensors-19-03685-s001.pdf]

sensors

Supplementary Materials: A High Voltage Energy Harvesting Interface for Irregular Kinetic Energy Harvesting in IoT Systems with 1365% Improvement using All-NMOS Power Switches and Ultra-low Quiescent Current Controller

Hassan Saif, Muhammad Bilawal Khan, Jongmin Lee, Kyoungho Lee and Yoonmyung Lee

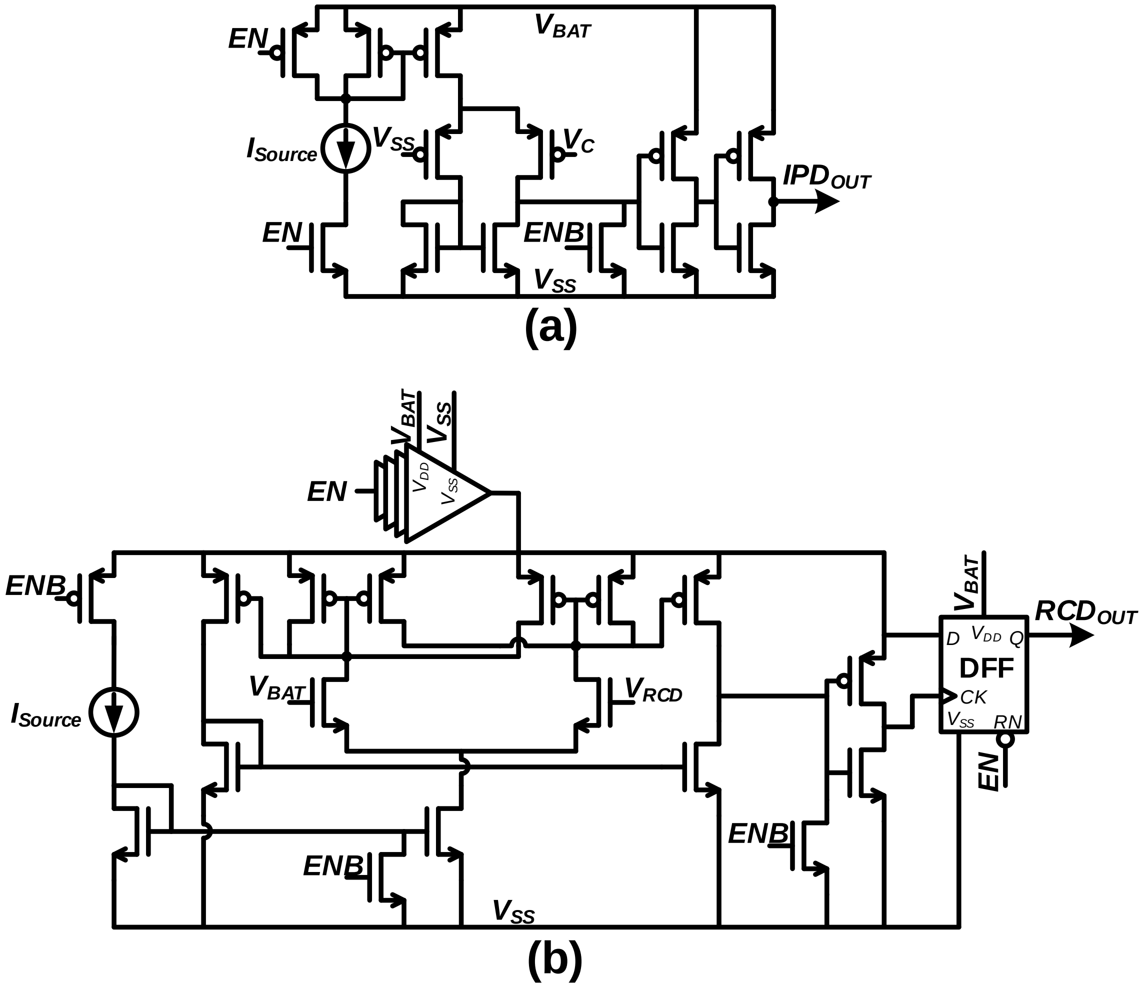

Figure S1. **(a)** Current peak detector (IPD) schematic. **(b)** Reverse current detector (RCD) schematic.

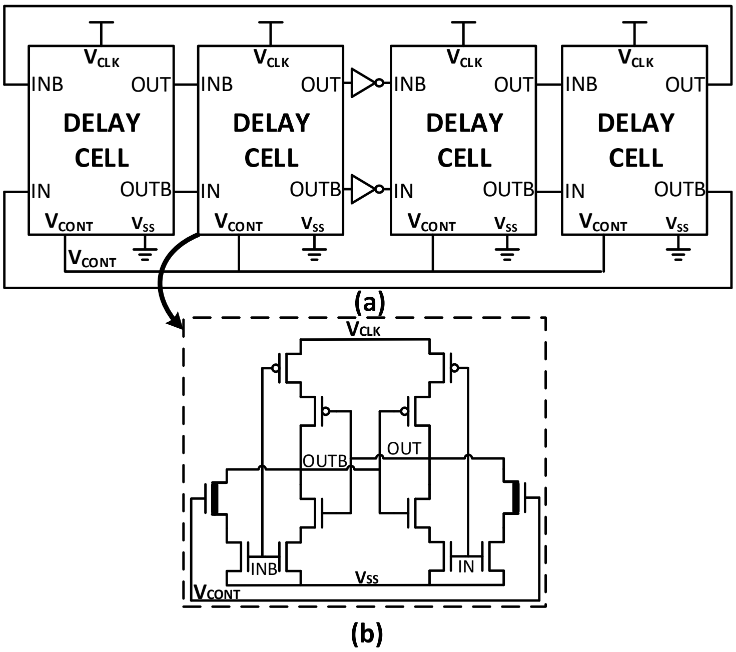

Figure S2. **(a)** Block diagram of leakage-based clock generator. **(b)** Symmetrical voltage controlled delay cell schematic.

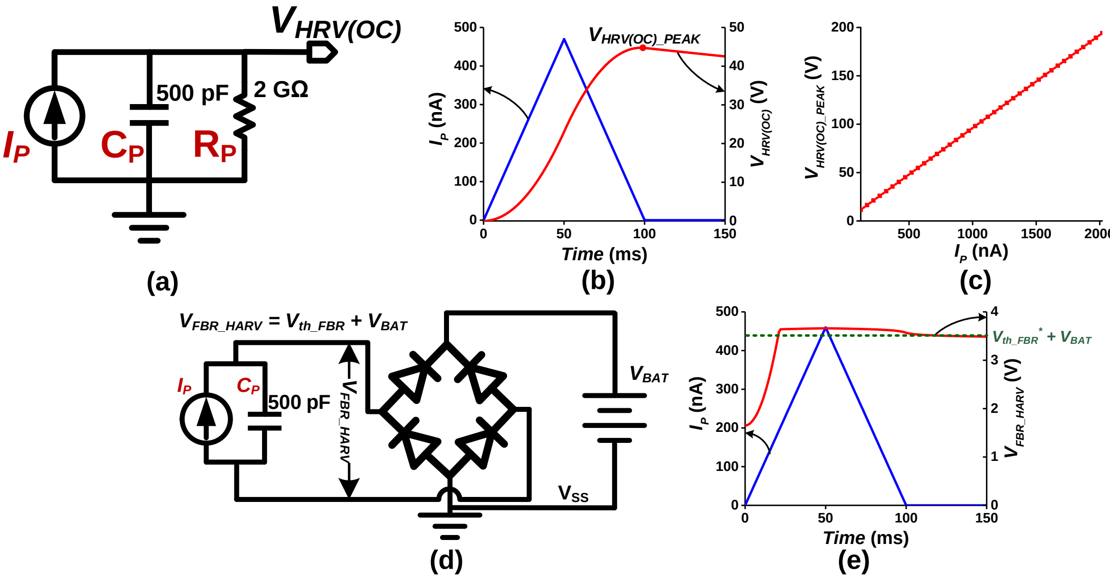

Figure S3. Flexible piezoelectric harvester

(a) Simulation model.

(b) Timing plot of input current triangular pulse (I_P) and open circuit harvester voltage (V_{HRV_OC}) for $V_{HRV(OC)\_PEAK}$ = *45 V*.

(c) Fixed period *(100ms)* I_P pulse vs V_{HRV_OC}.

(d) Simulation model for battery charging from flexible piezoelectric harvester using a full bridge rectifier (FBR).

(e) Timing plot of I_P vs FBR harvesting voltage (V_{FBR_HARV}) ($V_{th\_FBR}^*$ = FBR threshold voltage)

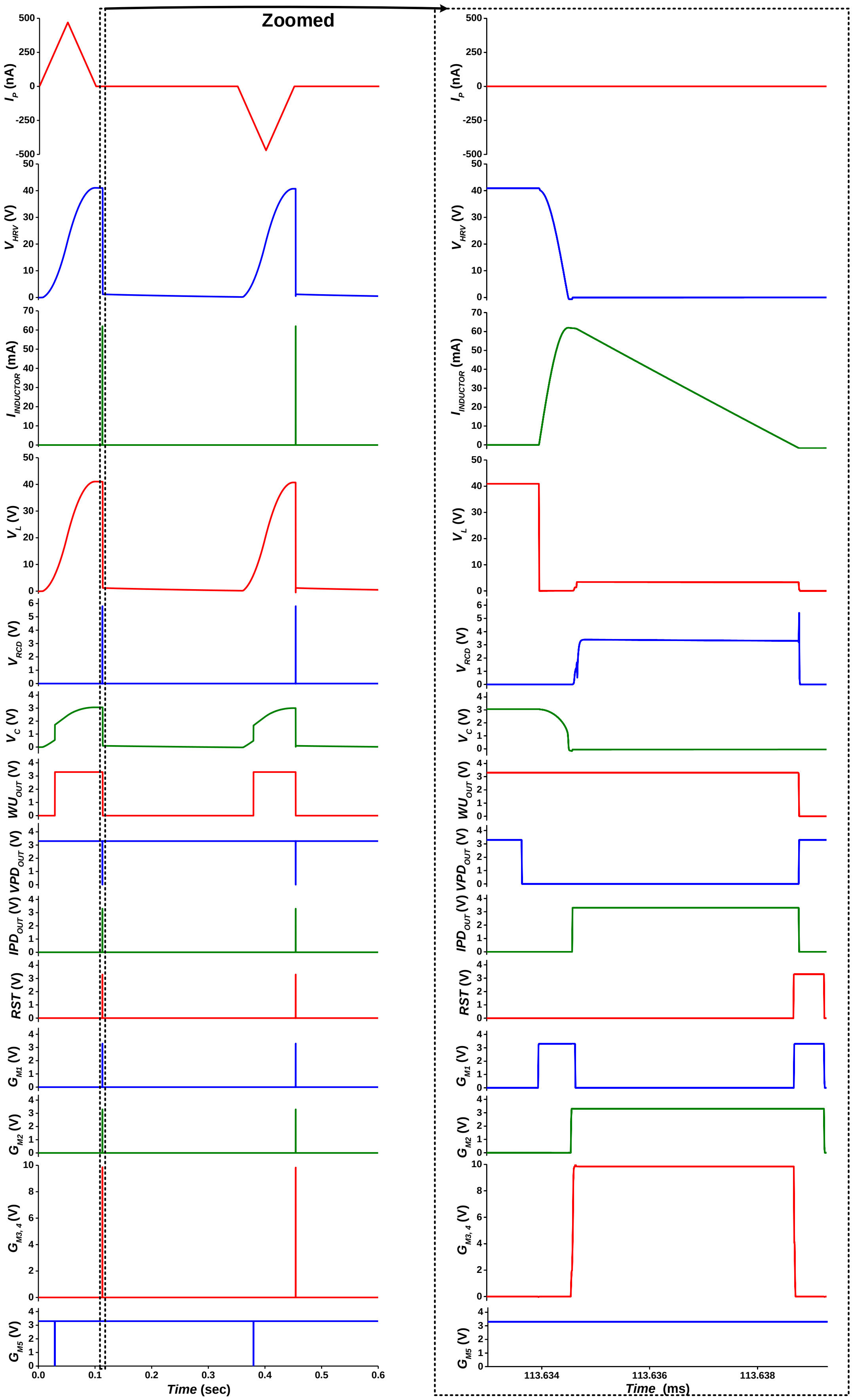

Figure S4. Proposed harvesting interface operation at charging *3.3V* battery at discontinuous harvesting from flexible piezoelectric harvester (C_P = 500pF) at $V_{HRV(OC)\_PEAK}$ = *45 V* .

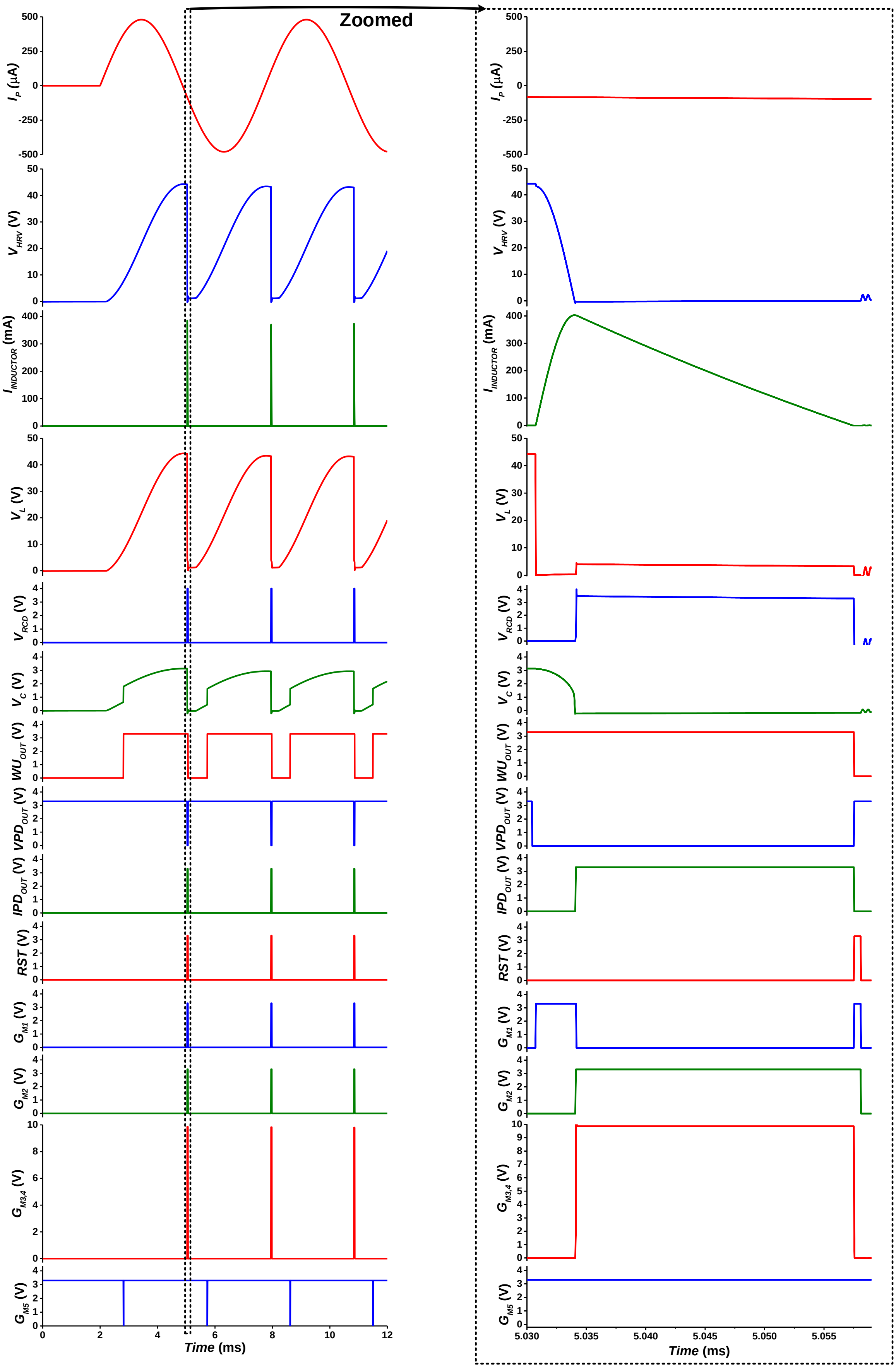

Figure S5. Proposed harvesting interface operation at charging *3.3V* battery at periodic harvesting from MIDE V22B harvester (C_P = 19.5nF) at $V_{HRV(OC)\_PEAK}$ = *45 V* .
